# A Genome-Wide RNAi Screen Identifies FOXO4 as a Metastasis-Suppressor through Counteracting PI3K/AKT Signal Pathway in Prostate Cancer

**DOI:** 10.1371/journal.pone.0101411

**Published:** 2014-07-01

**Authors:** Bing Su, Lingqiu Gao, Catherine Baranowski, Bryan Gillard, Jianmin Wang, Ryan Ransom, Hyun-Kyung Ko, Irwin H. Gelman

**Affiliations:** 1 Biomedical Research Institute, Shenzhen-PKU-HKUST Medical Center, Shenzhen, Guangdong, China; 2 Department of Cancer Genetics, Roswell Park Cancer Institute, Buffalo, New York, United States of America; 3 Department of Pharmacology and Therapeutics, Roswell Park Cancer Institute, Buffalo, New York, United States of America; 4 Department of Biostatistics and Bioinformatics, Roswell Park Cancer Institute, Buffalo, New York, United States of America; 5 Department of Pharmacology, Yale University School of Medicine, New Haven, Connecticut, United States of America; University of Kentucky College of Medicine, United States of America

## Abstract

Activation of the PI3K/AKT signal pathway is a known driving force for the progression to castration-recurrent prostate cancer (CR-CaP), which constitutes the major lethal phenotype of CaP. Here, we identify using a genomic shRNA screen the PI3K/AKT-inactivating downstream target, FOXO4, as a potential CaP metastasis suppressor. FOXO4 protein levels inversely correlate with the invasive potential of a panel of human CaP cell lines, with decreased mRNA levels correlating with increased incidence of clinical metastasis. Knockdown (KD) of FOXO4 in human LNCaP cells causes increased invasion *in vitro* and lymph node (LN) metastasis *in vivo* without affecting indices of proliferation or apoptosis. Increased Matrigel invasiveness was found by KD of FOXO1 but not FOXO3. Comparison of differentially expressed genes affected by FOXO4-KD in LNCaP cells in culture, in primary tumors and in LN metastases identified a panel of upregulated genes, including PIP, CAMK2N1, PLA2G16 and PGC, which, if knocked down by siRNA, could decrease the increased invasiveness associated with FOXO4 deficiency. Although only some of these genes encode FOXO promoter binding sites, they are all RUNX2-inducible, and RUNX2 binding to the PIP promoter is increased in FOXO4-KD cells. Indeed, the forced expression of FOXO4 reversed the increased invasiveness of LNCaP/shFOXO4 cells; the forced expression of FOXO4 did not alter RUNX2 protein levels, yet it decreased RUNX2 binding to the PIP promoter, resulting in PIP downregulation. Finally, there was a correlation between FOXO4, but not FOXO1 or FOXO3, downregulation and decreased metastasis-free survival in human CaP patients. Our data strongly suggest that increased PI3K/AKT-mediated metastatic invasiveness in CaP is associated with FOXO4 loss, and that mechanisms to induce FOXO4 re-expression might suppress CaP metastatic aggressiveness.

## Introduction

Prostate cancer (CaP) remains the most diagnosed non-cutaneous cancer and the second leading cause of cancer death in U.S. men [1]. The initial stages of CaP are regulated by androgen, thus, androgen deprivation therapy has been the mainstay of therapy for progressive prostate cancer. Most patients inevitably fail this therapy, progressing to castration-recurrent prostate cancer (CR-CaP) typically presenting as bone or lymph node metastases whose growth depends on sustained androgen receptor (AR) signaling [2]. Indeed, the targeting of CR-CaP with more specific anti-androgens or AR antagonists has offered significant, yet transient, clinical efficacy, and resistance still involves AR dependence, albeit involving AR mutants or overexpression [3]–[5].

Activation of the phosphatidylinositol-3-kinase (PI3K)/AKT pathway is a major contributor to CaP progression [6], [7] in that 42% of primary CaP lesions and 100% of metastatic tumors exhibit alterations (mutations/deletions, copy number variations, differential gene expression) in one or more components [8]. This has led to multiple clinical trials targeting PI3K, AKT or TORC1 in combination with standard chemotherapies (taxanes, platins) or antagonists of the androgen axis or AR [6]. Indeed, the prostate-specific loss of the PI3K/AKT antagonist, PTEN, in mouse transgenic models is sufficient to induce intraepithelial neoplasia [9], [10].

The FOXO family members, FOXO1, FOXO3a and FOXO4, are ubiquitously-expressed transcription factors that function as tumor suppressor proteins through their ability to repress the expression of genes encoding proliferative, survival or anti-differentiation functions [11], [12]. Roles for FOXO members in suppressing prostate cancer progression have been described. For example, FOXO1 deletion in 13q14 is associated with androgen- and AR-independent proliferation [13]. AKT, whose activity increases in CaP progression [7], directly phosphorylates FOXO family members, thereby antagonizing their function by promoting association with 14-3-3 proteins and preventing their nuclear translocation [14], leading to their ubiquitylation-mediated proteasome degradation [15]. The loss of FOXO3a promotes cancer formation in the TRAMP prostate cancer mouse model [16], whereas the upregulation or activation of FOXO proteins leads to growth arrest and apoptosis [17]–[19]. A study by Zhang et al. [20] demonstrates that FOXO1 inhibits CaP cell motility and invasiveness by preventing RUNX2 from binding to and transcriptionally activating progression genes such as *OP, IL8, VEGF* and *MMP13*. Although some redundant roles for FOXO proteins are implied by the finding that spontaneous thymic lymphomas and systemic hemangiomas are induced only upon the combined deletion of FOXO1, FOXO3 and FOXO4 [21], there is evidence from chromatin immunoprecipitation-sequencing (ChIP-seq) studies on FOXO1 and FOXO3a of both common and non-overlapping gene targets [22], [23]. For prostate cancer progression, the common or distinct roles for FOXO proteins remain unclear.

In order to identify genes that antagonize CaP metastasis, human LNCaP cells, which exhibit feeble invasiveness, were infected with a lentivirus-encoded genomic shRNA library and then selected for highly invasive cells in a Matrigel-coated Boyden chamber assay. Our data strongly suggest that FOXO4 suppresses metastatic invasiveness by preventing RUNX2 from activating a group of pro-metastatic genes including *PIP, PGC, CAMK2N1* and *PLA2G16*. The finding that FOXO4 downregulation correlates with earlier onset metastatic disease in CaP patients strongly suggests that CaP metastasis could be antagonized by reactivating FOXO4 expression or by inhibiting RUNX2 function.

## Materials and Methods

### Antibodies and reagents

The following primary antibodies (Ab) were used: rabbit polyclonals specific for FOXO1, FOXO3, FOXO4, cleaved caspase-3, GFP (Cell Signaling Technology, Beverly, MA); mouse monoclonals (mAb) include HA, GAPDH (Santa Cruz Biotechnology, Santa Cruz, CA), Myc (Applied Biological Materials, Richmond, BC, Canada), and Ki67 (Thermo Scientific/Pierce, Rockford, IL).

### Cell culture

LNCaP (ATCC CRL-1740) and CWR22Rv1 (ATCC CRL 2505) cells were cultured in RPMI1640 media supplemented with 10% FBS and incubated at 37°C in a humidified incubator containing 5% CO_2_. DU145 (ATCC HTB-81) and HEK293T (ATCC CRL-3216) cells were cultured in DMEM media supplemented with 10% FBS. LNCaP were infected with aliquots of the DECODE (OpenBiosystems) pooled pGIPZ lentivirus library encoding human genomic shRNAs (13,650 genes targeted in 7 pools of 9,750 shRNA clones/pool) at a multiplicity-of-infection of 1 (RPCI shRNA Core, Irwin Gelman, Director), and then selected for puromycin (2 :g/ml) resistance. Puromycin-resistant cells infected with empty pGIPZ alone served as a negative control.

### siRNA transfection

Synthetic ON-TARGETplus SMARTpool siRNA specific for FOXO4, FOXO1 and FOXO3, siCONTROL nonsilence siRNA (NS-siRNA), and DHarmaFECT-1 transfection reagent were purchased from Dharmacon (Lafayette, CO). LNCaP cells were plated at equal densities in 6-well plates (5×10^4^ per well) overnight. Cells were transfected with 50 nM of NS-siRNA or FOXO-specific siRNA for 24 h using DharmaFECT1 following the manufacturer's protocol.

### MTS cell growth assay

The proliferation of LNCaP cells stably infected with pGIPZ-FOXO4 shRNA or pGIPZ-NS-shRNA, or transiently transfected with FOXO4- or NS-siRNA was evaluated using MTS assay (Promega, Madison, WI) following the manufacturer's protocol. MTS assay measures the restoration of 3-(4, 5-dimethylthiazol-2-yl)-5-(3-carboxymethoxyphenyl)-2-(4-sulfophenyl)-2H-tetrazolium (MTS) to formazan by metabolically active cells.

### Immunoblot (IB) analysis

Cells were lysed in RIPA buffer (10 mM Tris, pH 7.4, 150 mM NaCl, 5 mM EDTA, 8% glycerol, 1% Triton X-100, 0.1% SDS, 0.5% sodium deoxycholate, 10 mM Na_3_VO4, 1 mM NaF, Complete Protease Inhibitor Cocktail (Roche Diagnostics, Mannheim, Germany)). 40 µg total protein/sample was separated by SDS-PAGE, blotted onto polyvinylidene fluoride membranes which were blocked for 30 min with 5% bovine serum albumin (Sigma) in 1×TBS/T (0.1% Tween-20 in Tris-buffered saline) and then probed as indicated. Digital imaging and signal quantification were performed on a Chemi-Genius^2^ Bio-Imager (Syngene, Frederick, MD) using GeneTools software.

### Transient transfection

pFOXO4-GFP or pFOXO4-TM-GFP, with Ala substitutions in all three AKT phosphorylation sites (kindly provided by Stefanie Dimmeler, University Frankfurt, Germany) were transiently transfected into CWR22Rv1. Myc-wtFOXO4 plasmid (kindly provided by Zhiping Liu, UT Southwestern Medical Center) was co-transfected transiently with pEGFP DNA (Clontech/Takara, Mountainview, CA) into CWR22Rv1 cells using Lipofectamine reagent (Invitrogen, Carlsbad, CA) according to the manufacturer's recommendations, and then incubated for 40h. GFP positive cells were scored for invasion and *in situ* zymography. For CHIP-qPCR analysis, HEK293T cells were transiently transfected with HA-RUNX2 (kindly provided by Jianmin Zhang, Roswell Park Cancer Institute), HA-RUNX2 plus Myc-FOXO4, or empty vector.

### Invasion assay and selection of invasive clones

Modified Boyden chamber assays were performed as previously described [24] starting with 5×10^4^ cells/5-well format. Values for migration were obtained by counting at least 10 cells in 6 fields per membrane (x20 objective) and averaged for three independent experiments. Cells with increased Matrigel invasiveness were isolated following four rounds of successive invasion assays. Specifically, invading cells (adhered to the bottom of transwell membranes) were removed by trypsinization, pooled based on shRNA modules, plated into 6-well dishes, and after expanding, re-subjected to invasion assays. After three rounds, cells were plated sparsely into 10 cm dishes, and after proliferation and colony isolation, bar codes were Sanger sequenced (RPCI Genomics Shared Resource Core, Irwin Gelman-Director) from isolated DNA using flanking PCR primer pairs, F:5'- ACGTCGAGGTGCCCGAAGGA-3' and R: 5'-AAGCAGCGTATCCACATAGCGT-3' or using the direct sequencing primer, 5′-GCATTAAAGCAGCGTATC-3′. LNCaP[vector] or LNCaP[shFOXO4] cells transiently transfected with 1 :g each of pGIPZ lentivirus shRNA clones (GFP-expressing) specific for PIP (accession #NM_002652.2; clones V2LHS170040 and V2LHS170041), CAMK2N1(accession #NM_018584.5, clones V2HS176164, V2HS176163, V2HS176165), PLA2G16 (accession #NM_007069.3, clonesV2LHS_253144, V2HS199589), PGC (accession #NM_002630.3, clone V2LHS169912) or RUNX2 (accession #NM_004348, clones V2LHS3151, V2LHS15062, V2LHS15065, V2LHS15066, V2LHS223856, V2LHS 224628), or empty pGIPZ vector control (OpenBiosystems) were assessed for invasive potential.

### *In situ* zymography

Glass coverslips were coated with 0.2 mg/ml Oregon Green 488-conjugated gelatin, cross-linked in 0.5% glutaraldehyde for 15 min at 4°C, and incubated with 5 mg/ml NaBH_4_ for 3 min. The coverslips were then disinfected with 70% ETOH for 15 min and washed in serum-free media for 1 h at 37°C. The cells were plated on coated coverslips, and incubated at 37°C for 24 h, fixed for 10 min with ice-cold 60% Acetone/3.7% paraformaldehyde in PBS, blocked with 3% non-fat dry milk in PBS for 30 min at RT. Myc-FOXO4 was stained with mouse anti-Myc (1∶500), and nuclei were stained with DAPI (Invitrogen; 1∶500), followed by FITC-conjugated goat anti-mouse IgG (1∶500; Chemicon, Temecula, CA). Fluorescent images were captured using a Nikon TE2000-E inverted microscope equipped with Roper CoolSnap HQ CCD camera. Invasiveness was quantified by measuring the average loss of FITC-gelatin area in triplicate slides.

### Orthotopic metastasis model

Orthotopic prostate injections into dorsal lobes of 10^6^ cells in 50 :l PBS in male nude mice embedded in their flanks with testosterone pellets was carried out as previously described [25]. After 10 weeks, mice were sacrificed and checked for GFP fluorescence (Lightools Research, Encinitas, CA) in primary tumors and in peripheral organs. All animal experiments were done under the supervision of the Institutional Animal Care and Use Committee of Roswell Park Cancer Institute under approved protocol 1177M. Anesthesia was inhaled Halothane to effect. Euthanasia was performed by CO2 asphyxiation followed by cervical dislocation.

### Immunohistochemistry (IHC)

Tissues were fixed in 10% buffered formalin for 24 h prior to processing. Fixed tissues were embedded in paraffin and sectioned at 5 :m. Slides were de-parafinized in xylenes and then rehydrated in graded alcohols followed by ddH_2_O. Slides were incubated in 1x pH 6 citrate buffer (Invitrogen Cat #00-5000) for 20 min and then in 3% H_2_O_2_ for 15 min. Slides were blocked with 10% normal goat serum for 30 min, followed by avidin/biotin block (Vector Labs Cat#SP-2001). Primary Abs to Ki67 (1∶500), GFP (1∶50) or caspase 3 (1∶200) were diluted in 1% BSA solution and incubated for 30 min at room temperature (RT), followed by incubation with biotinylated goat anti-rabbit secondary Ab (Cell Signaling Cat #9661) for 15 min. For signal enhancement, ABC reagent (Vector Labs Cat #PK 6100) was applied for 30 min, followed by incubation with DAB substrate (Dako Cat #K3467) for 5 min and counterstaining with modified Harris Hematoxylin (Richard-Allan Scientific Cat #72704) for 20 sec. Slides were washed extensively in PBS, sealed with coverslips and scanned in an Aperio ScanScope CS, using ImageScope software (Aperio Technologies, Inc., Vista, CA).

### Gene expression array

Total RNA was prepared using Trizol (Invitrogen) following manufacturer's instructions from six samples: LNCaP[shFOXO4] cell line, LNCaP[pGIPZ control] cell line, LNCaP[shFOXO4] primary tumor, LNCaP[pGIPZ control] primary tumor, LNCaP[shFOXO4] lymph node metastasis and LNCaP[pGIPZ control] lymph node metastasis. RNA samples were quantified using a ND-1000 spectrophotometer (Thermo Scientific/NanoDrop) and evaluated for degradation using a 2100 Bioanalyzer (Agilent Technologies, Santa Clara, CA). Samples with RNA Integrity Value (RIN) >7 were processed for gene expression array analysis using the Human HT-12 whole-genome gene expression beadchip array (v4) (Illumina, San Diego, CA). 500 ng of total RNA was converted to cDNA, followed by *in vitro* transcription to generate biotin labeled cRNA using the Ambion Illumina TotalPrep RNA Amplification Kit (Ambion/Life Technologies, Grand Island, NY) as per manufacturer's instructions. 750 ng of the labeled probes were then mixed with hybridization reagents and hybridized overnight at 58°C to the HT-12v4 BeadChips. Following washing and staining with Cy3-streptavidin conjugate, the BeadChips were imaged using an Illumina iScan Reader to measure fluorescence intensity at each probe. The intensity of the signal corresponds to the quantity of the respective mRNA in the original sample. BeadChip data files were analyzed with GenomeStudio (v2011.1; Illumina) gene expression module (v1.9.0) to report both un-normalized and quantile normalized, background-corrected gene expression signal levels. The gene expression signal levels were then analyzed using the Bioconductor packages Lumi and Limma programs (http://www.bioconductor.org). Genes with >2-fold expression changes were identified.

### Quantitative reverse transcriptase PCR (qRT-PCR)

Total RNA (1 µg/reaction) isolated using Trizol Reagent was subjected to reverse transcription reactions with random hexamer primers using the High Capacity cDNA Reverse Transcription kit (Life Technologies/Applied Biosystems). qRT-PCR was performed on a 7900HT Sequence Detection system (Life Technologies/Applied Biosystems) using FastStart Universal SYBR Green Master kit (Roche Diagnostics, Indianapolis, IN) following the manufacturer's instructions. The primer sequences (Table S2) were designed using Primer –BLAST (http://www.ncbi.nlm.nih.gov/tools/primer-blast/). GAPDH was used as a housekeeping gene for the qRT-PCR reactions. Each test was done in triple replication and the 2^−ΔΔCt^ method [26] was used to calculate the expression of genes.

### Co-immunoprecipitation (Co-IP) of FOXO4 and RUNX2

HEK293T cells were co-transfected with Myc-FOXO4 plus HA-RUNX2, HA-RUNX2 alone or empty vector alone. After 48 h, cells were lysed in RIPA buffer and IP was performed using HA Ab, followed by IB with the indicated Ab, or IP was performed using Myc Ab, followed by IB.

### Chromatin Immunoprecipitation (ChIP) –qPCR

ChIP assays were performed as previously described (9) with minor modifications. Briefly, LNCaP and HEK93T cells grown in 10-cm dishes (80–90% confluence) were crosslinked by adding 10% formaldehyde to culture media for 6–7 min at RT, followed by addition of glycine to 125 mM. Chromatin was sonicated to yield 100–300 bp fragments in SDS lysis buffer (0.1% SDS, 5 mM EDTA, 50 mM Tris-HCl, 150 mM NaCl, 2% NP-40, 0.5% deoxycholate, pH 8.1) containing 1 mM phenylmethanesulfonyl fluoride and protease inhibitor mixture. (Roche Applied Science) Following pre-clearing with protein A/G magnetic beads (Millipore), chromatin was incubated overnight at 4°C with 5 µg of the following Ab as indicated: HA, RUNX2, or normal mouse IgG. Immunocomplexes were pulled down with protein A/G magnetic beads. Crosslinks for both ChIP and input DNA were reversed at 65°C for 5 h and proteins were digested with proteinase K, and DNA was recovered by phenol/chloroform extraction and ethanol precipitation with 20 µg of glycogen as carrier as described [27]. Precipitated fragments were quantified by qPCR, and percentage input values were corrected for negative control regions where indicated. Primers for PCR amplification of PIP as previous described [28].

### Statistical analyses

Statistical significances between groups were determined by two-tailed student's t-test, with error bars signifying S.E.M. A p-value of <0.05 was considered significant.

## Results

### A genome-wide shRNA screen to identify candidate metastasis suppressor genes

**Figure 1:**
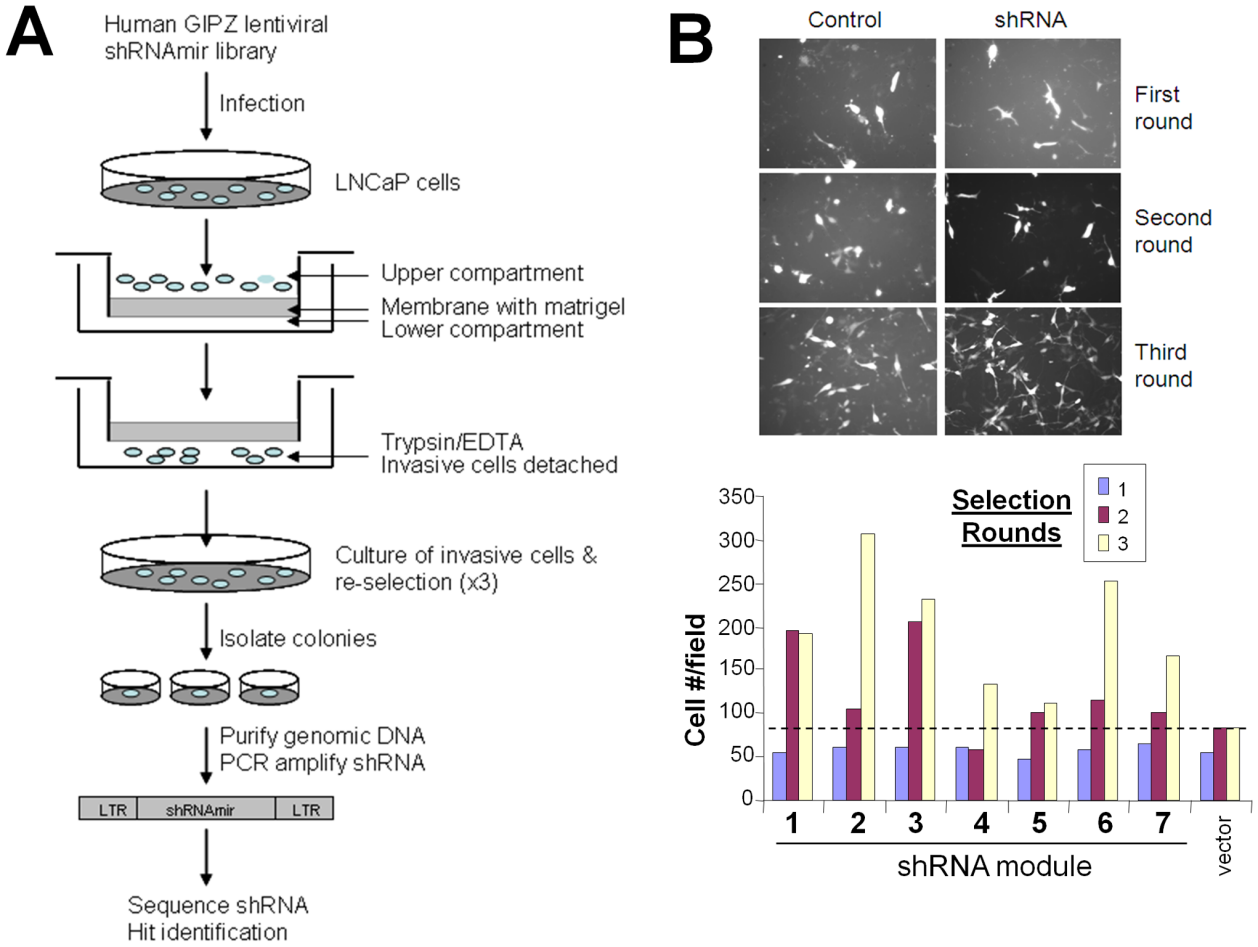
Loss-of-function selection for invasion-suppressing genes in LNCaP cells. **A.** Schematic summary of the screen. A high-throughput shRNA screening approach was used to identify genes whose knockdown induced tumor invasion, a process essential for metastasis. Highly invasive variants of LNCaP were selected using Matrigel-coated Boyden chamber invasion assays. **B.** Increased invasiveness induced by pools of shRNA clones over 3 rounds of selection. *Upper panel*: representative images of invasive GFP-expressing cells from control (pGIPZ vector) or shRNA (aliquot #2) after three selection rounds. *Lower panel*: invasive potential of pooled cells in each of 7 infection modules over three selection rounds, compared to LNCaP[vector] cells.

In order to understand the genetic components necessary for metastasis, we used a high throughput shRNA screening approach to identify genes capable of suppressing Matrigel invasiveness, a parameter of the metastatic cascade [29]. LNCaP cells, which exhibit low invasive potential [30], were infected with modules of pGIPZ (GFP-expressing) lentiviruses encoding modules of human genomic shRNAs at an MOI = 0.7 (to minimize cells transduced with multiple shRNAs), and after selection for puromycin resistance, cells were subjected to triplicate Matrigel Boyden chamber invasion assays. Invading cells (adhering to the bottom of the transwells membranes) were removed by trypsinization, pooled between triplicates, expanded in culture and then subjected to two more rounds of similar invasion assays. LNCaP infected with empty pGIPZ (LNCaP[vector] cells) were run in parallel to assess any selection of spontaneous increase in invasiveness (Fig. 1A). We assumed that cells with increasing invasiveness resulting from the loss of a suppressor function would be enriched with successive assay rounds. After three cycles of selection, colonies were isolated from modules 2, 3, 6, and 7, which showed increasing invasiveness relative to the Round 3 level of LNCaP[vector] controls (Fig. 1B). Sequence analysis (bar code and shRNA sequence) and BLAST databank searching (http://blast.ncbi.nlm.nih.gov/Blast.cgi) identified multiple clones of forkhead box O4 (*FOXO4*), kinesin family member 3B (*KIF3B*), signal transducing adaptor molecule (SH3 domain and ITAM motif) 1 (*STAM*), and Homo sapiens solute carrier family 17 (*SLC17A4*) (Table S1), together representing >94% of all the clones sequenced. Given the growing understand for roles for FOXO proteins as negative regulators of cancer progression [31], [32], and a recent study showing that up-regulation of ANXA8 by FOXO4 inhibits the cell migratory and metastatic characteristics of cholangiocarcinoma cells [33], we focused on the possible role of FOXO4 as a potential metastasis suppressor.

### Down-regulation of FOXO4 in human metastatic CaP cell lines and metastatic tissues

**Figure 2:**
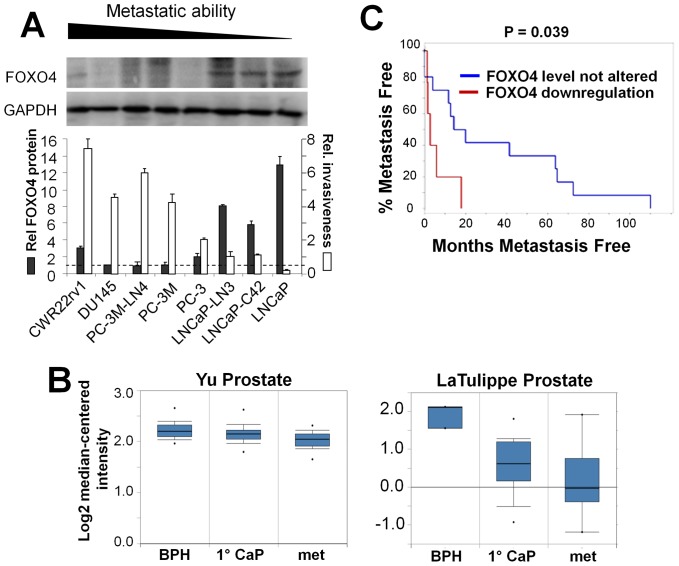
FOXO4 expression in human CaP cell lines and metastatic tissues. **A.** IB analysis of FOXO4 expression in human CaP cell lines (*upper panel*), quantified and compared to relative Matrigel invasiveness in the *lower panel*. Error bars, SE of three independent IB analyses quantified by densitometry (for FOXO4 levels) or Matrigel invasion assays. The relative FOXO4 level in DU145 was set at a value = 1 (dotted line). **B.** Oncomine studies FOXO4 RNA expression levels in BPH, primary-site (1°) CaP or metastases (mets) from LaTulippe et al. [45] and Yu et al. [46]. **C.** Kaplan-Meier plot analysis (http://www.cbioportal.org/public-portal/) of metastasis occurrence vs. time-to-onset in 37 CaP metastasis cases from Taylor et al. [8] in which 12 cases (32%) displayed FOXO4 downregulation and correlated with a more rapid appearance of metastases compared with the 25 cases that showed no changes in FOXO4 expression levels.

To test whether FOXO4 gene expression correlates with CaP cell invasiveness, we first compared FOXO4 protein expression in a panel of CaP cell lines with differing invasive and metastatic potentials. There was an inverse correlation between Matrigel invasiveness and FOXO4 protein levels (Fig. 2A), especially when comparing LNCaP to two metastatic variants, LN3 and C4-2. A search of the Oncomine database (http://www.oncomine.org) revealed that FOXO4 was significantly down-regulated in metastatic CaP compared to primary-site cancer and/or normal samples from two individual studies (Fig 2B). Furthermore, analysis of metastatic CaP cases from the study of Taylor et al. [8] in the *cbio* website (http://www.cbioportal.org/public-portal/) showed that those with FOXO4 downregulation correlated with a more rapid appearance of metastases compared with those with no altered FOXO4 expression (Fig. 2C). There was no evidence correlating FOXO4 downregulation in breast cancer metastasis although there was a small downregulation in colon cancer metastasis (Fig. S1), suggesting that any putative metastasis suppressor function by FOXO4 would be tissue-type specific. FOXO1 levels were also downregulated in CaP metastasis (Fig. S2A), however, we could not determine whether FOXO1 loss correlated with increased metastasis in CaP patients (Fig. S2B) because of underpowering due to low patient numbers. In contrast, FOXO3 expression levels showed no correlation with CaP metastasis (Figs. S2C&D).

### FOXO4 loss in LNCaP CaP cells contributes to highly metastatic potential

**Figure 3:**
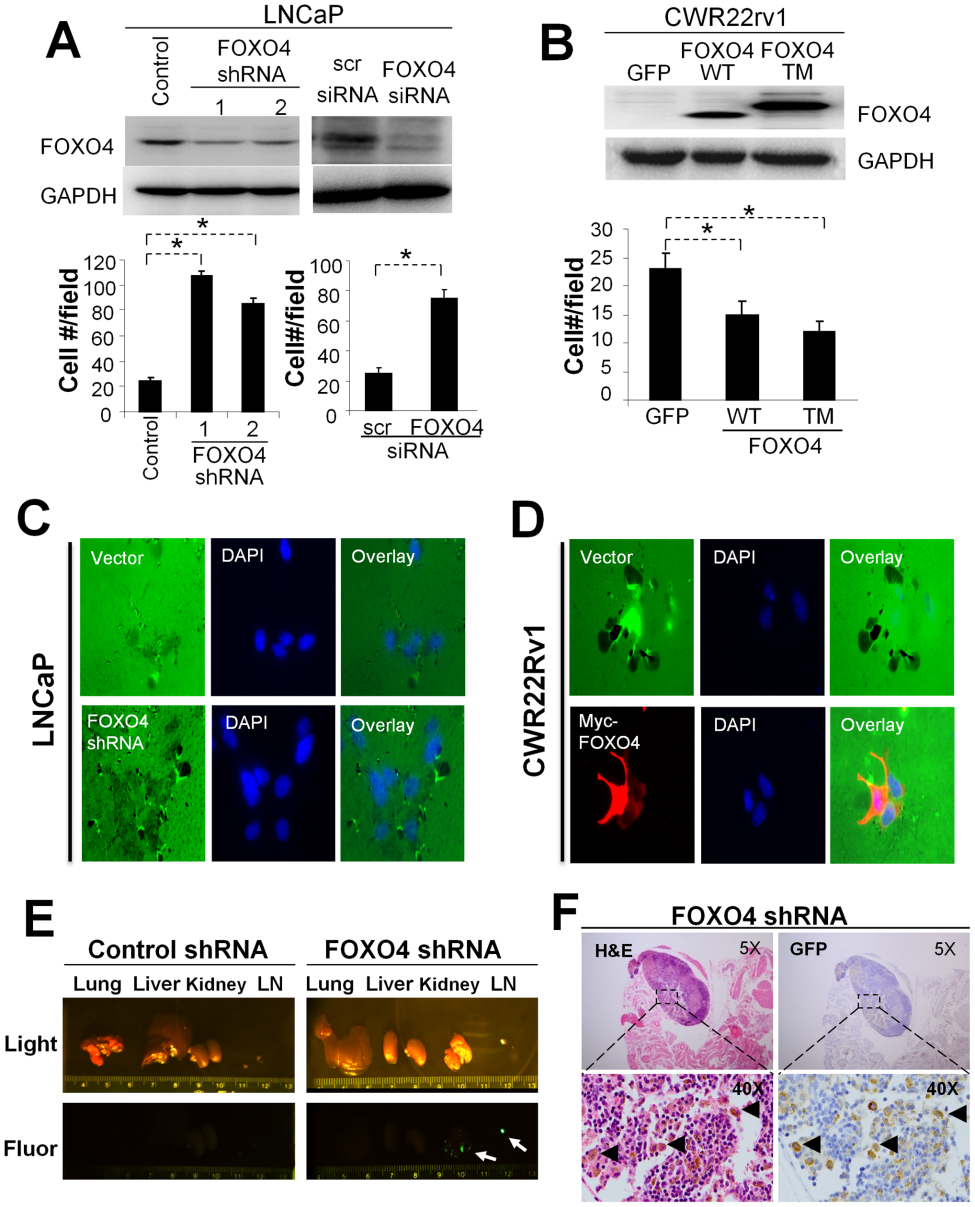
FOXO4 regulates invasiveness *in vitro* and metastasis *in vivo*. **A.** LNCaP cells stably transduced with shRNA or transiently transfected with siRNA against FOXO4 (vs. scrambled control) were tested for invasiveness. FOXO4 knockdown was assessed by IB (*upper panel*) and its effect on Matrigel invasiveness was quantified (*lower panel*). Error bars, S.E. of triplicate experiments. *, p<0.01. **B.** Ectopic expression of WT or constitutively-active (TM) FOXO4 decreases CWR22Rv1 invasiveness. Ectopic FOXO4 was assessed by IB (*upper panel*) and its effect on Matrigel invasiveness was quantified (*lower panel*). Error bars, S.E. of triplicate experiments. *, p<0.01. **C.** The local invasiveness of LNCaP[vector] (*upper row*) vs. LNCaP[shFOXO4] (*lower row*) was assessed for cells seeded onto Oregon Green 488-labeled gelatin, with cells labeled by DAPI. **D.** The same analysis as in **C** except comparing CWR22Rv1 stably expressing vector or Myc-FOXO4. **E.** Lung, liver, kidney and LN from individual mice tumored with LNCaP[vector] (control) or LNCaP[shFOXO4] were imaged using visible light (*upper panel*) or fluorescent light (*lower panel*). Arrows, LN and kidney metastases. **F.** Metastatic LN lesions from a LNCaP[shFOXO4] tumored mouse stained for H&E or GFP (by IHC). Triangles, examples of GFP-positive metastatic cells.

To determine whether down-regulation of FOXO4 contributed to the increased invasive capacity of LNCaP cells, LNCaP cells were stably transduced with lentivirus FOXO4 shRNA clones, different from the one identified in the original screen, or transfected with siFOXO4, and these cells, vs. vector or scrambled (scr) siRNA controls, were tested for invasiveness. The knockdown of FOXO4 by sh- or siFOXO4 resulted in 2.5- to 4-fold increases in LNCaP invasiveness (Fig. 3A). Conversely, CWR22Rv1 cells transiently cotransfected with pEGFP plus either wt-FOXO4 or a constitutively-active FOXO4 mutant (TM, for “triple mutant”, i.e.- loss of all three AKT phosphorylation sites) resulted in decreased invasiveness (Fig. 3B). Loss of FOXO4 from shRNA or siRNA had no effect on LNCaP proliferation rates in 2D cultures (Fig. S3A) suggesting that increased invasion levels after FOXO4 were not due to changes in proliferation rates. In addition, we analyzed how FOXO4 controls the invasiveness of CaP cells seeded onto Oregon Green 488-labeled gelatin-coated cover slips. FOXO4 knockdown in LNCaP resulted in increased localized digestion of Oregon Green 488-labeled gelatin extracellular matrix compared to cells expressing non-specific shRNA (Fig. 3C) whereas the overexpression of Myc-FOXO4 in CWR22Rv1 cells decreased localized digestion (Fig. 3D).

Given that the FOXO family members share some redundant functions [31], we analyzed FOXO1 and FOXO3 levels in CaP cells and tested their ability to control invasiveness. In contrast to FOXO4, FOXO1 and FOXO3 levels seemed to increase in the more invasive variants of LNCaP and PC-3 cells (Figs. S4A&B). Interestingly, the knockdown of FOXO1, but not FOXO3, in LNCaP cells induced higher invasiveness (Fig. S4C), yet the forced expression of either FOXO1 or FOXO3 failed to decrease the invasiveness of CWR22Rv1 cells (Fig. S4D). Thus, whereas FOXO1 may be involved with LNCaP invasiveness, FOXO4 is both involved with and sufficient for the control of invasiveness.

We then tested whether FOXO4 knockdown could promote spontaneous metastasis *in vivo*. Male nude mice embedded with time-release testosterone pellets were injected in their dorsal lobes with LNCaP[vector] or LNCaP[shFOXO4] cells, and after ten weeks, the mice were sacrificed and analyzed for GFP fluorescence as a marker of pGIPZ. Although the LNCaP[shFOXO4] primary-site tumors were slightly smaller than those induced by LNCaP[vector] cells, this difference was not statistically significant (Fig. S3B). Importantly, there was no difference in their relative GFP expression (Fig. S3C), or their proliferation or apoptosis rates *in vivo* based on Ki67 or cleaved caspase-3 IHC staining (Figs. S3D&E, respectively). FOXO4 knockdown resulted in the increased incidence of GFP-expressing macrometastases to local draining lymph nodes (LN), kidney (Fig. S3E) and lung compared to control cells (Table 1). Specifically, 82% (9/11) of the shFOXO4 group had LN metastases, compared to 31% (5/16) for the control group; 27% (3/11) and 9.1% (1/11) in the shFOXO4 group had kidney and lung metastases, respectively, whereas metastases were found in none (0/16) of the control group. In addition, we confirmed that the LN lesions expressed GFP protein using GFP-specific IHC (Fig. 3F).

**Table 1:** Incidence of macro-metastasis.

Group	Tumor	Macro-metastasis (GFP+)	
		Pelvic Lymph node	Lung
Control (n = 20)	16/20 (80%)	5/16 (31%)	0/16 (0%)
FOXO4 ShRNA (n = 20)	11/20 (55%)	9/11 (82%)	1/11 (9.1%)*

*Also had pelvic lymph node metastasis.

### Analysis of FOXO4-regulated pro-metastasis genes

**Figure 4:**
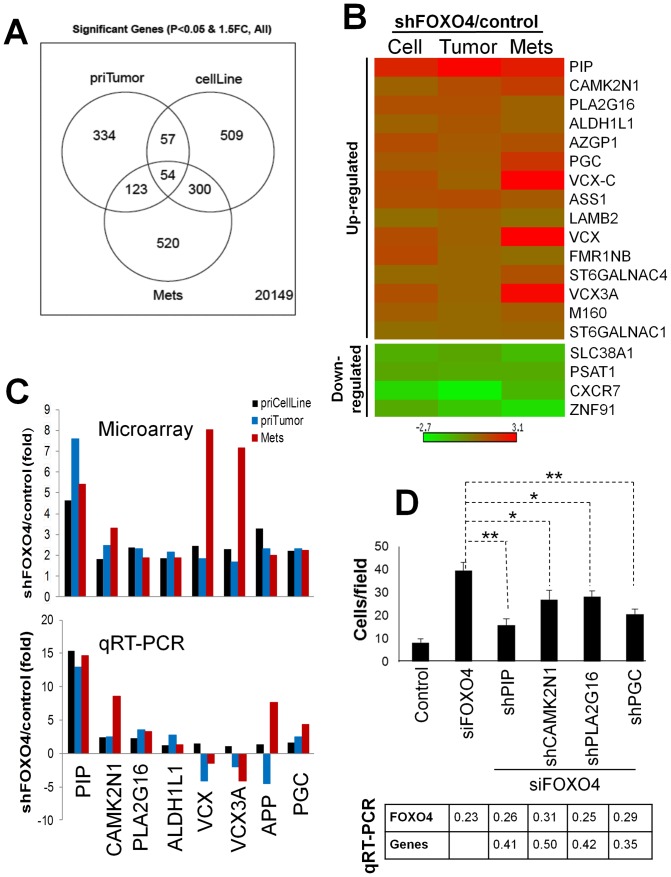
Identification of candidate pro-metastasis genes regulated by FOXO4. **A.** Venn diagram showing unique and commonly genes differentially expressed after FOXO4 knockdown in LNCaP cells (“cellLine”), primary-site tumors (“priTumor”) and KN metastases (“mets”). **B.** Heat map of common up- and downregulated genes differentially expressed after FOXO4 knockdown. **C.** Eight genes commonly upregulated in expression microarrays (*upper panel*) after FOXO4 knockdown (*PIP, CAMK2N1, PLA2G16, ALDH1L1, VCX, VCX3A, APP*, and *PGC*) were analyzed by qRT-PCR (*lower panel*). **D.** LNCaP co-transfected with siFOXO4 or scrambled siRNA (control) plus shRNAs specific for the 4 upregulated genes validated in **C**, were subjected to Matrigel invasion assays (*upper panel*). Error bars, S.E. of triplicate experiments. *, P<0.05; **, P<0.01. The relative knock of each gene was confirmed by qRT-PCR relative to non-specific shRNA controls (*lower panel*).

Given that FOXO proteins function as transcriptional repressors, we explored whether the increased invasiveness after FOXO4 loss might be due to the increase in pro-metastasis gene expression. Thus, genome-wide gene expression analysis was performed on LNCaP[vector] vs. LNCaP[shFOXO4] cultured cells, primary-site tumors and LN metastases. Knockdown of FOXO4 in each of these sample sets was confirmed by IB (Fig. S5A). This analysis identified 535 genes whose expression changed ≥1.5-fold (Fig. S5B). Of these, 54 changes were common to all three samples sets (Fig. 4A, Fig. S5C), and of these, 19 genes (15 upregulated, 4 downregulated) were associated with FOXO4 knockdown (Fig. 4B). In order to focus our analysis, we analyzed by Ingenuity IPA software which of the 19 gene signature was involved in metastasis-associated processes such as cell motility, invasiveness, chemotaxis or cell survival. Eight genes up-regulated in shFOXO4-associated LN metastases (*PIP, CAMK2N1, PLA2G16, ALDH1L1, VCX, VCX3A, APP*, and *PGC*) were identified as potentially involved in metastasis (Fig. 4C, *top*). Of these, only *PIP, CAMK2N1, PLA2G16* and *PGC* were confirmed by qRT-PCR as consistently increased after FOXO4 knockdown in LNCaP culture cells, primary tumors and LN metastases (Fig. 4C, *bottom*).

We assumed that the upregulation of some of these genes facilitates the increased invasiveness detected after FOXO4 knockdown. To test this, we co-transfected LNCaP cells with siRNA to FOXO4 plus plasmid constructs (pGIPZ) encoding PIP-, CAMK2N1-, PLA2G16- or PGC-specific shRNAs and then monitored for invading GFP-expressing cells. siFOXO4 transduction resulted in 3- to 4-fold reduction in FOXO4 levels, and the gene-specific shRNAs resulted in 2- to 3-fold reductions, as assessed by qRT-PCR (Fig. 4D, *bottom*). The loss of *PIP* or *PGC*, and to a lesser extent, *CAMK2N1* or *PLA2G16*, blunted the enhanced invasiveness of LNCaP induced by the loss of *FOXO4* (Fig. S4D, *top*). These data strongly suggest that FOXO4 normally suppresses invasiveness by inhibiting the expression of PIP, CAMK2N1, PLA2G16 and PGC.

### FOXO4 regulates metastasis through control of the PI3K/AKT downstream target, RUNX2

**Figure 5:**
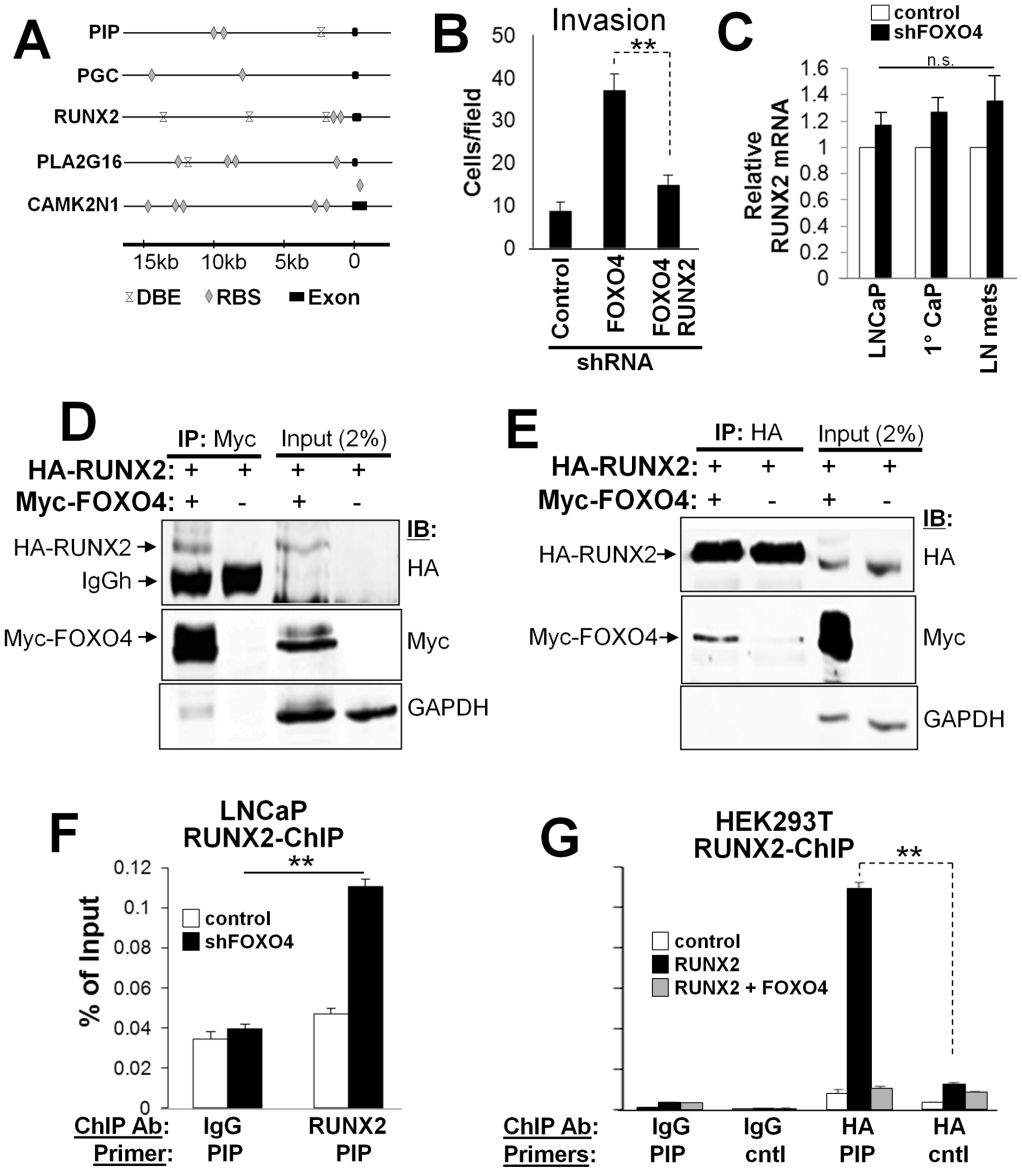
FOXO4 regulates metastasis by binding to and suppressing RUNX2 transactivation ability. **A.** Promoter regions of *RUNX2, PIP, PGC, PLA2G16* and *CAMK2N1* showing potential FOXO (DBE) and RUNX2 (RBS) binding sites relative to first exons. **B.** Matrigel invasion assay of LNCaP cells expressing control, FOXO4 or FOXO4 plus RUNX2 shRNAs. Error bars, S.E. of triplicate experiments. **, P<0.02. **C.** Relative RUNX2 RNA levels, as assessed by qRT-PCR in control shRNA vs. shFOXO4 LNCaP cells, primary tumors or LN metastases. RNA levels in each control condition were set to 1. Error bars, S.E. of triplicate experiments. n.s., not significant. Lysates of HEK293T cells transfected with HA-RUNX2 and Myc-FOXO4 were either analyzed by IB for HA, Myc or GAPDH, or immunoprecipitated with anti-myc and blotted with anti-HA (**D**), or immunoprecipitated with anti-HA and blotted with anti-Myc (**E**). **F.** Chromatin from LNCaP[vector] (control) or LNCaP[shFOXO4] cells were immunoprecipitated with control IgG or RUNX2 Ab, and the precipitated DNA subjected to qPCR using PIP promoter primers (Table S2). Error bars, S.E. of triplicates. **, P<0.01. **G.** Chromatin from HEK293T cells transfected with expression plasmids for RUNX2, RUNX2+FOXO4 or empty vector were immunoprecipitated with control IgG or HA Ab, then analyzed for PIP DNA by qPCR as in **F**.

We next addressed whether the upregulation of PIP, CAMK2N1, PLA2G16 and PGC after FOXO4 knockdown was due to the loss of FOXO4's repressive activity. A scan of 15 Kb of promoter regions for the FOXO4 DNA binding element, TTGTTTAC (DBE) [34] indicated that *PIP* and *PLA2G16*, but not *PGC* and *CAMK2N1*, promoters encoded potential sites for FOXO4 interaction (Fig. 5A). In order to determine whether FOXO4 bound to any of these, LNCaP were transfected with HA-tagged FOXO4-TM, and after ChIP of FOXO4 (vs. Ig control), the precipitated DNA was amplified by PCR using either DBE-flanking primers or primers flanking non-DBE control regions. Our data indicated that FOXO4 did not bind to the putative DBE in the *PIP* and *PLA2G16* promoters (not shown). This suggests that the increase in PIP, CAMK2N1, PLA2G16 and PGC after FOXO4 knockdown is not due to a direct regulation by FOXO4.

It is noteworthy that *PIP* and *PGC* were identified previously as RUNX2-regulated targets [35], [36], and that *PIP, PGC, CAMK2N1* and *PLA2G16* share multiple potential RUNX2 binding sites (Fig. 5A) based on the motif, (T/A/C)G(C/T)GGT [37]. RUNX2 has been shown to play a key role in prostate cancer metastasis, but mostly in regards to crosstalk between CaP and bone cells during development of osteoblastic metastases [37]–[39]. Moreover, increased nuclear staining of RUNX2 was an independent marker of metastatic disease in human CaP [40]. This suggests that RUNX2 is an antagonist of FOXO4, and indeed, RUNX2 knockdown in LNCaP[shFOXO4] cells blunted their enhanced Matrigel invasiveness (Fig. 5B). Although the RUNX2 promoter has three putative FOXO4 DBE (Fig. 5A), RUNX2 mRNA levels were relatively unchanged by FOXO4 knockdown in LNCaP cells, primary tumors or LN metastases (Fig. 5C), suggesting that FOXO4 does not antagonize RUNX2 by altering its expression level. Based on the recent demonstration that FOXO1 inhibits CaP cell migration and invasiveness by binding to and inhibiting RUNX2 transcriptional activity [20], we addressed whether FOXO4 affects RUNX2 function through protein-protein interaction. To confirm this, lysates of HEK293T cells transfected with HA-RUNX2 and Myc-FOXO4 were subjected to HA-specific IP followed by MYC IB. Our results show RUNX2 co-precipitation with FOXO4 in reciprocal co-IP experiments (Fig. 5D&E). Furthermore, although decreased FOXO4 did not alter RUNX2 levels (Fig. 5C), there was increased RUNX2 binding in LNCaP[shFOXO4] vs. control cells, as shown by ChIP-qPCR, to a PIP promoter site (Fig. 5F) previously shown to facilitate transcriptional activation by RUNX2 [35]. In agreement with this finding, the increased ability of ectopic HA-tagged RUNX2 to bind to the PIP promoter in HEK293T cells was antagonized by co-expression of FOXO4 (Fig. 5G). Taken together, these data strongly suggest that FOXO4 controls expression of pro-metastasis genes, such as PIP, by directly inhibiting RUNX2 transactivation activity.

**Figure 6:**
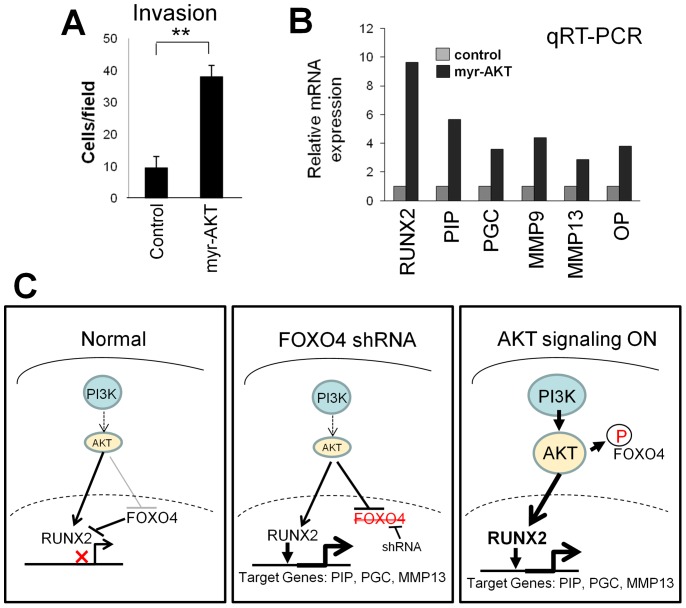
Control of invasiveness and pro-invasion RUNX2-regulated genes by AKT. **A.** Ectopic expression of activated AKT (myr-AKT) increases LNCaP cell invasion. Error bars, S.E. of triplicate experiments. **, P<0.01. **B.** Relative mRNA levels of *RUNX2*, and RUNX2-regulated genes, *PIP, PGC, MMP9, MMP13* and *OP*, assessed by qRT-PCR, in LNCaP cells stably expressing myr-AKT or an empty vector control. **C.** Model for PI3K/AKT negative regulation of FOXO4 and RUNX2 in the context of expression control of pro-invasion target genes.

Activation of the PI3K/AKT axis in CaP [7] likely leads to FOXO4 inactivation through its direct phosphorylation by AKT, resulting in its retention in the cytosol [31]. In contrast, activated AKT plays a direct role in activating RUNX2, thereby facilitating its role in promoting metastasis [37]. Therefore, we addressed how activated AKT could drive LNCaP invasiveness through the activation of RUNX2-regulated genes. The stable expression of constitutively-activated AKT (myr-AKT) increased the invasion of LNCaP cells (Fig 6A). Moreover, activated AKT induced the expression of RUNX2 and several of its target genes, such as *PIP, PGC, MMP9, MMP13* and *OP* (Fig. 6B). These data strongly suggest that CaP metastasis is promoted by the ability of AKT to inhibit FOXO4 nuclear function, including its antagonism of RUNX2, and by inducing RUNX2 expression and the expression of RUNX2-induced, pro-metastasis genes (Fig. 6C). In contrast, the forced decrease of FOXO4 is sufficient to induce RUNX2 and gene targets, thereby increasing metastatic invasiveness.

## Discussion

The current study identifies FOXO4 as a potentially novel metastasis suppressor amongst several candidate genes identified using a genomic shRNA screen for increased LNCaP invasiveness. FOXO4 likely fulfills the currently accepted definition of a metastasis suppressor [41] in that it is downregulated in clinical metastases compared to primary-site CaP lesion, its downregulation correlates with significantly decreased time-to-onset of clinical metastasis, its expression levels do no grossly affect primary tumor growth, yet its downregulation promotes metastatic invasiveness *in vitro* and metastastic formation *in vivo*. Although redundant functionality between FOXO family members is known [21], only FOXO4 exhibited critical regulatory ability for invasiveness, namely that FOXO4 knockdown resulted in increased invasiveness whereas its overexpression suppressed invasiveness. Given this unique function for FOXO4, and that our evidence suggests that FOXO4 regulates metastasis by suppressing the ability of RUNX2 to induce pro-metastasis genes (discussed below), it is likely that CaP metastasis is promoted by a non-redundant FOXO4 cistrome and/or by unique interactions between the repressor function of FOXO4 and pro-metastasis transcription factors.

The known role of FOXO family members, including FOXO4, as transcriptional repressors led us to analyze FOXO4-regulated gene expression changes shared by cultured LNCaP, primary tumors and LN metastases. Our assumption is that a gene signature arising after FOXO4 knockdown would identify functions that contributed to increased invasiveness *in vitro*, and that if this signature was maintained through primary tumor cells and into metastases, these functions would likely also control increased metastatic potential. Of the initial 19 genes identified by gene expression microarrays that fulfilled this definition, a 4-gene signature, *PIP, PGC, CAMK2N1* and *PLA2G16*, passed qRT-PCR validation and, as well, showed evidence in the literature of involvement with metastatic processes. The notion that FOXO4 might directly repress expression of this gene group was ruled out because i) only two gene promoters, those of *PIP* and *PLA2G16*, encode FOXO binding sites, and ii) overexpressed FOXO4 did not bind these sites in ChIP-qPCR experiments. However, bioinformatics analysis of these genes and promoters indicated that they were all RUNX2-targets and -regulated genes, and indeed, RUNX2 knockdown could blunt the enhanced invasiveness induced by the loss of FOXO4 in LNCaP cells. Moreover, we showed that RUNX2 and FOXO4 interacted in cells, and that altering FOXO4 levels had an inverse effect on the ability of RUNX2 to bind its cognate site on the *PIP* promoter yet did not change RUNX2 expression levels. This finding parallels that of Zhang et al. [20], who showed that FOXO1 binding to RUNX2 suppressed invasiveness by preventing RUNX2 access to the promoters of pro-metastasis genes such as *OP, IL8, VEGF* and *MMP13*.

A role for RUNX2 in promoting crosstalk between CaP and osteoblasts/osteoclasts in bone metastases has been known for some time, especially in the context of being an inducer of genes regulating extracellular matrix proteolysis, osteolysis, and tumor cell epithelial-to-mesenchymal transition [39]. Importantly, increased nuclear localization of RUNX2 correlates with increased CaP metastasis and poorer outcomes [40], strengthening the notion that RUNX2 is a metastasis promoter in CaP. A recent study [36] demonstrates that RUNX2 synergizes with AR to promote CaP invasiveness through the upregulation of pro-metastasis genes such as *PIP, PGC* and *SNAI1*. Indeed, the majority of promoter/enhancer sites occupied by RUNX2 in CaP cells identify genes that drive CaP invasiveness and membrane trafficking/secretion functions [42].

The FOXO4/RUNX2 antagonism we identified continues to function downstream of PI3K/AKT signaling. Specifically, activated AKT1 (encoded in the myr-AKT used here) was sufficient to induce LNCaP invasiveness and concomitant increases in *RUNX2* levels and in RUNX2 pro-metastasis target genes, *PIP, PGC, MMP9, MMP13* and *OP*. Thus, the known increase in AKT activation levels during CaP malignancy progression [6] would likely lead to inactivation of FOXO4 due to a direct phosphorylation and cytoplasmic sequestration, resulting in increased RUNX2 transcriptional activity. Indeed, AKT1 activation (myr-AKT1) is sufficient to induce increased DU145 Matrigel invasiveness [43] and CaP metastasis in a transgenic model lacking TGFβRII [44]. These data strongly suggest that AKT kinase inhibitors or antagonists of RUNX2 transcriptional activity would be capable of preventing or treating CaP metastases by inhibiting expression of pro-metastasis genes.

## Supporting Information

Figure S1 FOXO4 mRNA levels in breast and colon cancer. Comparison of FOXO4 mRNA levels in normal, primary-site (1°) tumor and metastases in the Bittner breast cancer (http://www.ncbi.nlm.nih.gov/geo/query/acc.cgi?acc=GSE2109) and Ki colon cancer studies (http://www.ncbi.nlm.nih.gov/geo/query/acc.cgi?acc=GSE6988) as described in the Oncomine database (http://www.oncomine.org).
(TIF)

Figure S2 The expression of FOXO1/3 in prostate cancer. Oncomine data showing downregulated FOXO1 (A) or FOXO3 (C) RNA expression levels in metastatic CaP in studies by LaTulippe et al. and Yu et al. (see Fig. 2). Data from Taylor et al. analyzed in *cbio* (see Fig. 2) showing no statistical significance between downregulation of FOXO1 (B) or FOXO3 (D) with increased time-to-onset of metastasis in CaP patients.
(TIF)

Figure S3 Knockdown of FOXO4 has no obvious effect on tumor growth and apoptosis in LNCaP/FOXO4 shRNA orthotopic nude mouse model. **A**. Proliferation of LNCaP cells stably infected with lentiviruses encoding pGIPZ-FOXO4shRNA or scrambled (scr)-shRNA, or transiently transfected with FOXO4- or non-specific (NS)-siRNA was evaluated by MTS assay. GFP expression in tumor cells (**B**) showing no significant difference in tumor size induced by LNCaP[vector] or LNCaP[shFOXO4] cells (**C**). LNCaP[vector] and LNCaP[shFOXO4] tumors stained by IHC for Ki67 (**D**) or cleaved caspase-3 (**E**). *Right panels*: Quantification of stained cells. Error bars, S.D. of stained cells in 6 microscopic fields at x20 magnification. Neither showed statistical significance (n.s.).
(TIF)

Figure S4 Invasion of LNCaP cells after knockdown of FOXO1 or FOXO3. **A**. IB analysis of FOXO1 and FOXO3 expression in human CaP cell lines (GAPDH levels as protein loading control). **B.** Graphic representation of the data in Panel A, normalized to the FOXO1 levels in DU145 cells equal to 1 (hatched line). Error bars, S.E. of two independent experiments. LNCaP cells (**C**) transiently transfected with siRNA against FOXO1 or FOXO3 vs. control (NSC, non-specific control) or CWR22Rv1 cells (**D**) transiently transfected with pEGFP alone or with FOXO1 or FOXO3 expression vectors were tested for Matrigel invasiveness. *Upper panels*: IB of FOXO1 or FOXO3 protein levels (vs. GAPDH controls). *Lower panels*: Matrigel invasion assay. Error bar, S.D.; *, p<0.01. n.s., no significance.
(TIF)

Figure S5 Identification of candidate FOXO4-regulated genes by using RNA microarray analysis. **A.** Knockdown of FOXO4 in each set sample was confirmed by IB. **B.** Heat-map representing unsupervised hierarchical clustering of expression values of genes from all samples. **C.** The list of common FOXO4-regulated gene expression changes in all three sample sets (LNCaP cell line, primary tumor and LN metastasis).
(TIF)

Table S1 Candidate metastasis suppressor genes identified in shRNA screen.
(TIF)

Table S2 Primers used in this study.
(TIF)
