# Supplementary figures and images for: A Genome-Wide RNAi Screen Identifies FOXO4 as a Metastasis-Suppressor through Counteracting PI3K/AKT Signal Pathway in Prostate Cancer
Source: PLoS One. 2014 Jul 1;9(7):e101411. doi: 10.1371/journal.pone.0101411 (PMC4077825; doi:10.1371/journal.pone.0101411)

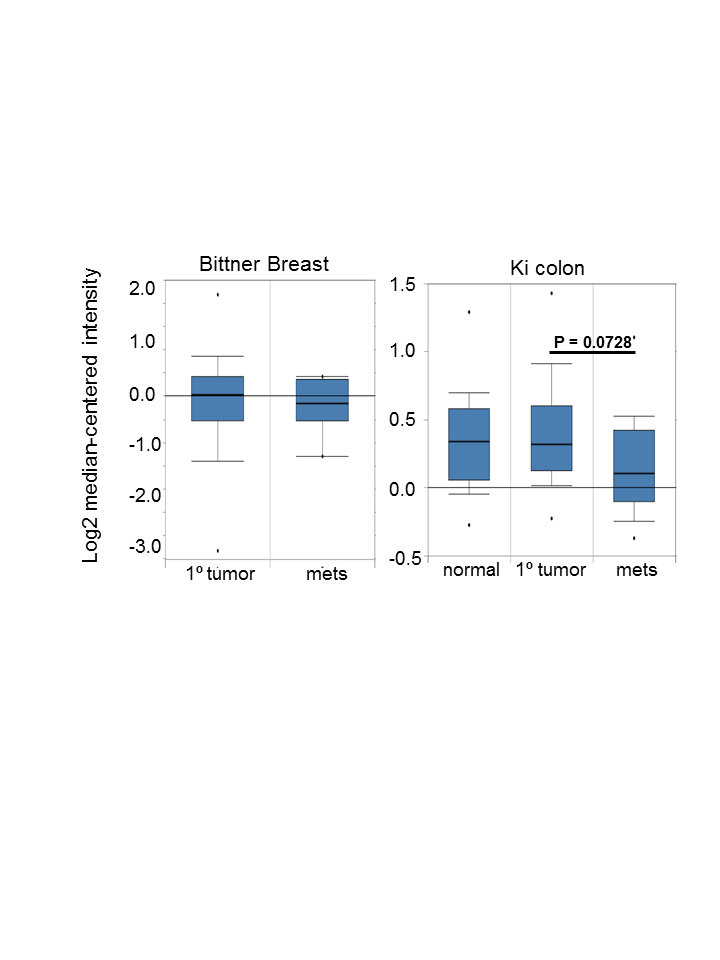

Supplement: Figure S1 — FOXO4 mRNA levels in breast and colon cancer. Comparison of FOXO4 mRNA levels in normal, primary-site (1°) tumor and metastases in the Bittner breast cancer (http://www.ncbi.nlm.nih.gov/geo/query/acc.cgi?acc=GSE2109) and Ki colon cancer studies (http://www.ncbi.nlm.nih.gov/geo/query/acc.cgi?acc=GSE6988) as described in the Oncomine database (http://www.oncomine.org). (TIF) [file pone.0101411.s001.tif]

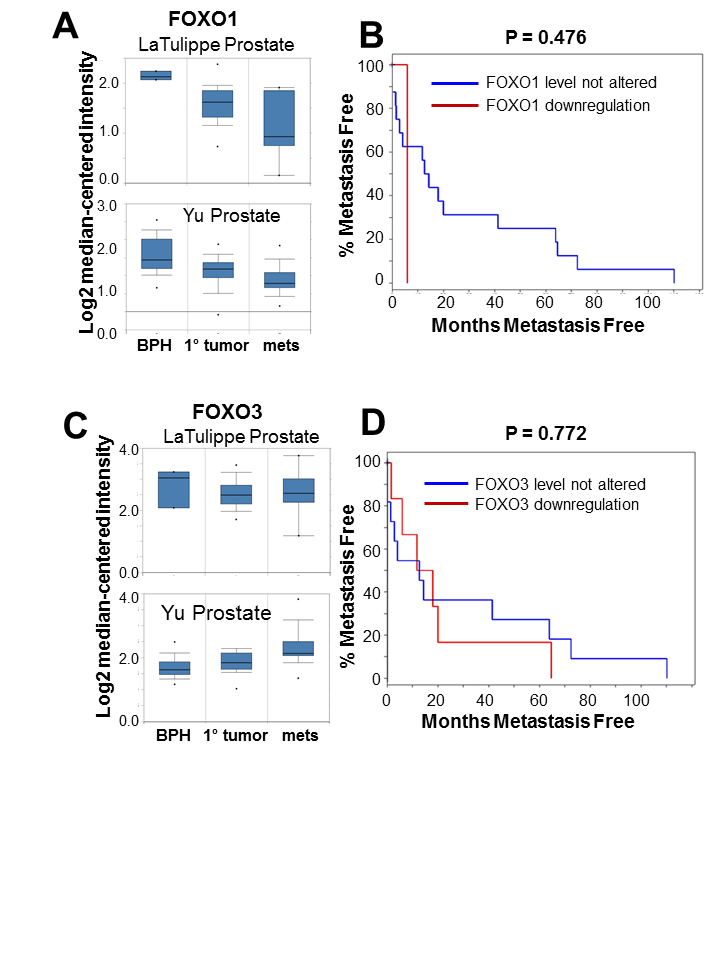

Supplement: Figure S2 — The expression of FOXO1/3 in prostate cancer. Oncomine data showing downregulated FOXO1 (A) or FOXO3 (C) RNA expression levels in metastatic CaP in studies by LaTulippe et al. and Yu et al. (see Fig. 2). Data from Taylor et al. analyzed in cbio (see Fig. 2) showing no statistical significance between downregulation of FOXO1 (B) or FOXO3 (D) with increased time-to-onset of metastasis in CaP patients. (TIF) [file pone.0101411.s002.tif]

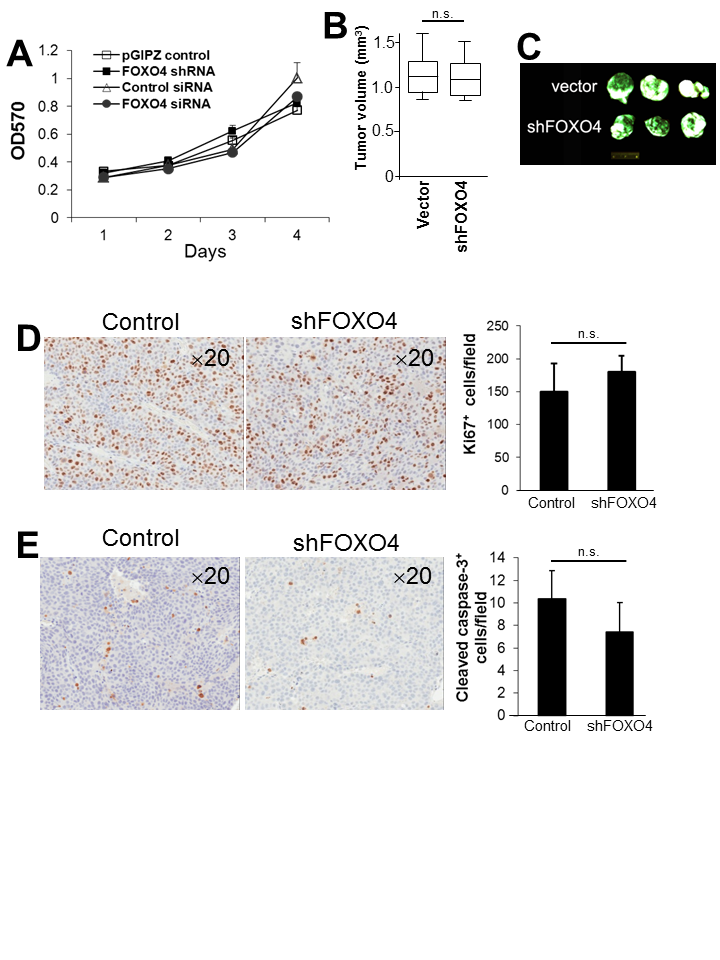

Supplement: Figure S3 — Knockdown of FOXO4 has no obvious effect on tumor growth and apoptosis in LNCaP/FOXO4 shRNA orthotopic nude mouse model. A. Proliferation of LNCaP cells stably infected with lentiviruses encoding pGIPZ-FOXO4shRNA or scrambled (scr)-shRNA, or transiently transfected with FOXO4- or non-specific (NS)-siRNA was evaluated by MTS assay. GFP expression in tumor cells (B) showing no significant difference in tumor size induced by LNCaP[vector] or LNCaP[shFOXO4] cells (C). LNCaP[vector] and LNCaP[shFOXO4] tumors stained by IHC for Ki67 (D) or cleaved caspase-3 (E). Right panels: Quantification of stained cells. Error bars, S.D. of stained cells in 6 microscopic fields at x20 magnification. Neither showed statistical significance (n.s.). (TIF) [file pone.0101411.s003.tif]

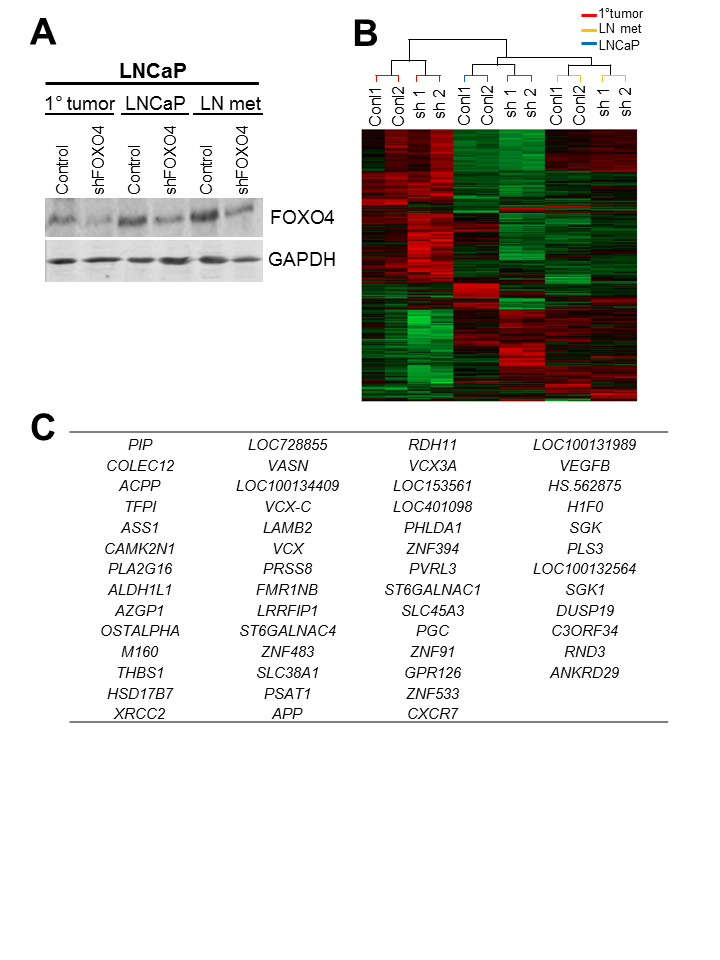

Supplement: Figure S5 — Identification of candidate FOXO4-regulated genes by using RNA microarray analysis. A. Knockdown of FOXO4 in each set sample was confirmed by IB. B. Heat-map representing unsupervised hierarchical clustering of expression values of genes from all samples. C. The list of common FOXO4-regulated gene expression changes in all three sample sets (LNCaP cell line, primary tumor and LN metastasis). (TIF) [file pone.0101411.s005.tif]
